# Biochemical and molecular features of Chinese patients with glutaric acidemia type 1 detected through newborn screening

**DOI:** 10.1186/s13023-021-01964-5

**Published:** 2021-08-03

**Authors:** Yiming Lin, Wenjun Wang, Chunmei Lin, Zhenzhu Zheng, Qingliu Fu, Weilin Peng, Dongmei Chen

**Affiliations:** 1 Center of Neonatal Disease Screening, Quanzhou Maternity and Children’s Hospital, 700 Fengze Street, Quanzhou, 362000 Fujian Province China; 2 Hangzhou Biosan Clinical Laboratory, Hangzhou, 310007 Zhejiang Province China; 3 Department of Neonatology, Quanzhou Maternity and Children’s Hospital, 700 Fengze Street, Quanzhou, 362000 Fujian Province China

**Keywords:** Glutaric acidemia type 1, newborn screening, *GCDH* gene, free carnitine, primary carnitine deficiency

## Abstract

**Background:**

Glutaric acidemia type 1 (GA1) is a treatable disorder affecting cerebral organic acid metabolism caused by a defective glutaryl-CoA dehydrogenase (*GCDH*) gene. GA1 diagnosis reports following newborn screening (NBS) are scarce in the Chinese population. This study aimed to assess the acylcarnitine profiles and genetic characteristics of patients with GA1 identified through NBS.

**Results:**

From January 2014 to September 2020, 517,484 newborns were screened by tandem mass spectrometry, 102 newborns with elevated glutarylcarnitine (C5DC) levels were called back. Thirteen patients were diagnosed with GA1, including 11 neonatal GA1 and two maternal GA1 patients. The incidence of GA1 in the Quanzhou region was estimated at 1 in 47,044 newborns. The initial NBS results showed that all but one of the patients had moderate to markedly increased C5DC levels. Notably, one neonatal patient with low free carnitine (C0) level suggest primary carnitine deficiency (PCD) but was ultimately diagnosed as GA1. Nine neonatal GA1 patients underwent urinary organic acid analyses: eight had elevated GA and 3HGA levels, and one was reported to be within the normal range. Ten distinct *GCDH* variants were identified. Eight were previously reported, and two were newly identified. In silico prediction tools and protein modeling analyses suggested that the newly identified variants were potentially pathogenic. The most common variant was c.1244-2 A>C, which had an allelic frequency of 54.55% (12/22), followed by c.1261G>A (p.Ala421Thr) at 9.09% (2/22).

**Conclusions:**

Neonatal GA1 patients with increased C5DC levels can be identified through NBS. Maternal GA1 patients can also be detected using NBS due to the low C0 levels in their infants. Few neonatal GA1 patients may have atypical acylcarnitine profiles that are easy to miss during NBS; therefore, multigene panel testing should be performed in newborns with low C0 levels. This study indicates that the *GCDH* variant spectra were heterogeneous in this southern Chinese cohort.

**Supplementary Information:**

The online version contains supplementary material available at 10.1186/s13023-021-01964-5.

## Background

Glutaric acidemia type 1 (GA1; OMIM #231670) is a treatable disorder affecting cerebral organic acid metabolism and is caused by a defective glutaryl-CoA dehydrogenase (*GCDH*) gene. This gene encodes the glutaryl-CoA dehydrogenase (GCDH, EC 1.3.8.6) that mediates the degradation of L-lysine, L-hydroxylysine, and L-tryptophan [1]. GA1 leads to the accumulation of glutaric acid (GA) and 3-hydroxyglutaric acid (3HGA) in bodily tissues, particularly in the brain. The clinical presentation of GA1 varies, ranging from the more common infantile-onset disease (3 months to 6 years) to the less common later-onset disease (older than 6 years). Untreated GA1 patients typically present with acute encephalopathy, movement disorders (MD), and striatal damage in the first 3–36 months [2]. GA1 patients are divided into low (LE) and high excretors (HE), based on the amount of urinary GA. However, both biochemical subtypes are at similar risk of developing MD if left untreated [3–5].

Elevated glutarylcarnitine (C5DC) levels can be reliably detected in GA1 patients using tandem mass spectrometry (MS/MS), and early therapy has proven to be effective. Therefore, GA1 has been added to various national newborn screening (NBS) panels [6, 7]. Previous investigations demonstrated that NBS for GA1 has significantly improved the neurologic outcome of affected individuals. Early identification through NBS is essential in providing timely treatment and ensuring optimal outcomes [8]. However, data on the incidence, NBS experience, and mutational spectrum in China is limited. We encountered one confirmed GA1 case but with a normal C5DC profile at initial screening. Additionally, two mothers with GA1 were incidentally diagnosed using our NBS program. Herein, we report the acylcarnitine profiles and molecular features of 13 GA1 patients diagnosed via NBS in a southern Chinese population.

## Results

### NBS for GA1

A total of 102 newborns had abnormal C5DC concentrations at initial NBS, yielding a positivity rate of 0.02% (102/517,484). Ten newborns with elevated C5DC levels were diagnosed with GA1, with a positive predictive value (PPV) of 9.8% (10/102). Surprisingly, one newborn with a low C0 level was also diagnosed with GA1. Consequently, the incidence of GA1 in the Quanzhou region was estimated to be 1 in 47,044 newborns. In addition, two mothers were diagnosed with GA1 based on the abnormal NBS results of their infants.

### Biochemical features

The initial NBS results showed that all but one of the patients had moderate to markedly increased C5DC levels. This patient (no. 7) had a normal C5DC level and an extremely low C0 level during NBS. Mean C5DC concentrations during NBS and the recall stage in the neonatal GA1 patients were 2.40 ± 1.08 µmol/L and 2.17 ± 1.28 µmol/L (reference value: 0.03–0.3 µmol/L), respectively. Notably, two mothers were found to have GA1 based on the low C0 levels of their infants. Both mothers had elevated C5DC levels and low C0 levels. Nine neonatal GA1 patients underwent urinary organic acid analyses: eight had elevated GA and 3HGA levels (HE phenotype), and one was reported within the normal range (LE phenotype) (Table 1).

**Table 1:** Biochemical and genetic features of 11 neonatal GA1 patients

Patient no.	Gender	Age at diagnosis (days)	Newborn screening		Recall		Urine organic acids		Genotype^a^	
			C5DC (µmol/L)	C0 (µmol/L)	C5DC (µmol/L)	C0 (µmol/L)	GA (mmol/mol creatinine)	3HGA (mmol/mol creatinine)	Allele 1	Allele 2
1	F	22	2.67	13.50	2.47	15.06	150.49	9.88	c.532G>A (p.Gly178Arg)	c.108_109delAC (p.Gln37Glufs*5)
2	F	20	1.45	18.32	1.50	41.12	503.79	23.36	c.533G>A (p.Gly178Glu)	c.1244-2 A>C^b^
3	F	18	2.81	13.95	3.68	10.87	300.15	30.98	c.1244-2 A>C	c.1244-2 A > C
4	M	25	2.42	13.06	N/A	N/A	409.05	36.34	c.1244-2 A>C	c.1244-2 A>C
5	F	20	1.90	14.25	1.92	10.20	462.83	12.91	c.395G>A (p.Arg132Gln)	c.1147 C>T (p.Arg383Cys)
6	F	21	3.79	13.33	N/A	N/A	N/A	N/A	c.1244-2 A>C	**c.1016T>C (p.Met339Thr)**
7	F	44	0.06	**3.18**	0.05	**7.69**	1.31	2.50	c.1244-2 A>C	c.1261G>A (p.Ala421Thr)
8	M	23	1.58	16.37	2.19	29.91	313.74	24.96	c.1244-2 A>C	c.1244-2 A>C
9	M	28	4.26	9.66	3.34	4.99	741.81	65.23	c.1244-2 A>C	c.1244-2 A>C
10	F	26	2.44	10.91	3.65	9.04	434.34	38.46	c.1244-2 A>C	c.1261G>A (p.Ala421Thr)
11	F	30	0.68	19.51	0.70	20.62	N/A	N/A	c.300G>A (p.Met100Ile)	c.1204 C>T (p.Arg402Trp)

M: male, F: female, C5DC: glutarylcarnitine; C0: free carnitine, GA: glutaric acid, 3HGA: 3-hydroxyglutaric acid, N/A: not available. Reference range, C5DC: 0.03–0.3 µmol/L, C0: 8.5–50 µmol/L, GA: < 2.5 mmol/mol creatinine, 3HGA:  <4.6 mmol/mol creatinine

^a^The novel variants identified by our team are in boldface type

^b^The c.1244-2 A>C splice site variant destroys the canonical splice acceptor site in intron 11 of the *GCDH* gene, and is expected to cause aberrant splicing

### Genetic testing and variants analysis

All 11 neonatal GA1 patients harbored compound heterozygous or homozygous variants in *GCDH*. Ten distinct variants were identified, eight of which were previously reported pathogenic variants. The two remaining variants were newly identified by our team. The most common variant was c.1244-2 A>C, which had an allelic frequency of 54.55% (12/22), followed by c.1261G>A (p.Ala421Thr) (9.09%, 2/22). Similarly, two maternal GA1 patients had biallelic pathogenic variants in *GCDH*, and their infants were only carriers (Table 2).

**Table 2:** Biochemical and genetic features of two maternal GA1 patients and their infants

No.^a^	Gender	Current age (years)	Newborn screening		Recall		Urine organic acids		Affected gene	Genotype	
			C5DC (µmol/L)	C0 (µmol/L)	C5DC (µmol/L)	C0 (µmol/L)	GA (mmol/mol creatinine)	3HGA (mmol/mol creatinine)		Allele 1	Allele 2
1	Female	37	1.07	4.00	1.15	5.92	579.6	29.11	*GCDH*	c.1244-2 A>C	c.1244-2 A>C
2	Male	5	0.07	4.12	0.04	5.84	N/A	N/A	*GCDH*	c.1244-2 A>C	
3	Female	34	0.76	2.46	N/A	N/A	N/A	N/A	*GCDH*	c.1063 C>T (p.Arg355Cys)	c.769 C>T (p.Arg257Trp)
4	Male	3	0.06	3.68	0.03	7.16	N/A	N/A	*GCDH*	c.769 C>T (p.Arg257Trp)	

C5DC: glutarylcarnitine; C0: free carnitine, GA: glutaric acid, 3HGA: 3-hydroxyglutaric acid, N/A: not available

Reference range, C5DC: 0.03–0.3 µmol/L, C0: 8.5–50 µmol/L, GA: < 2.5 mmol/mol creatinine, 3HGA: < 4.6 mmol/mol creatinine

^a^No. 1 and 3 are maternal GA1 patients, No. 2 and 4 are their infants, respectively

**Fig. 1:**
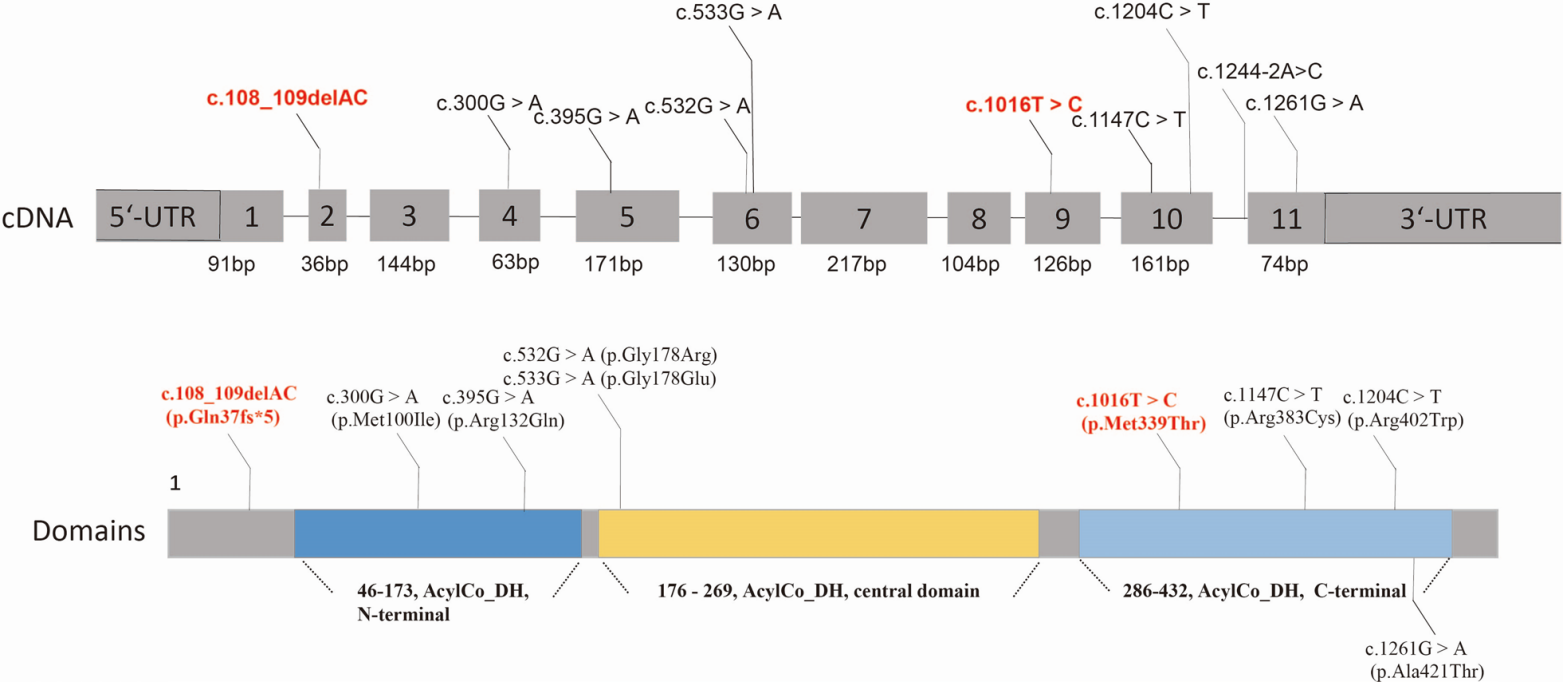
*GCDH* variant spectra and Glutaryl-CoA dehydrogenase domains (The novel variants identified by our team are highlighted in red; AcylCo_DH represent Acyl-CoA dehydrogenase)

**Fig. 2:**
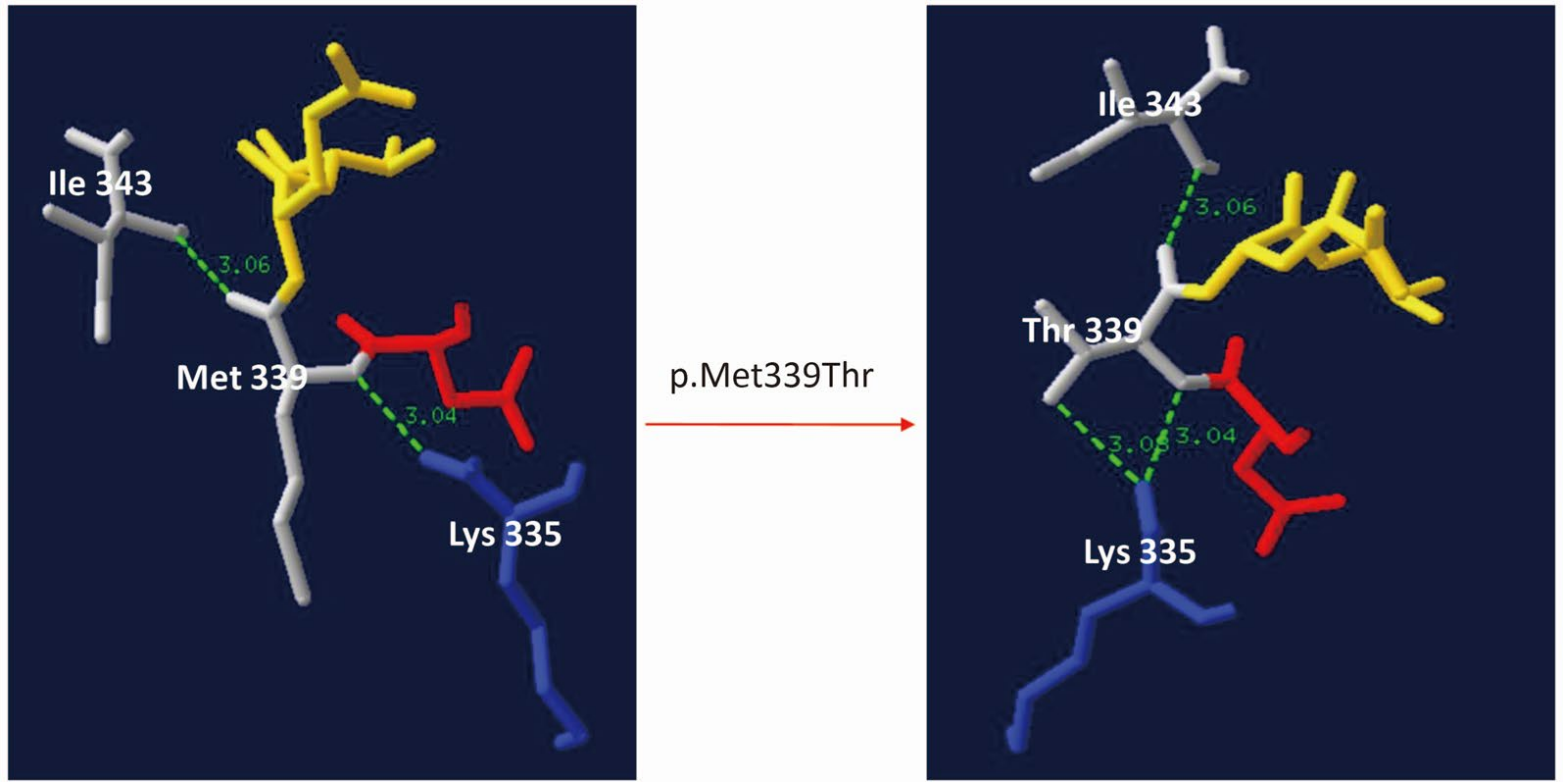
Three-dimensional modeling structure analysis of wild-type and mutant products of GCDH. Green dashed lines represent hydrogen bonds, and the green number indicates hydrogen bond distances. Protein modeling showed that variant c.1016T>C (p.Met339Thr) increased hydrogen bonds between the protein position 335 and 339, which may affect the structure and function of the GCDH protein by altering the folding process

The newly identified variants have not been described in human genome variation databases such as dbSNP, GnomAD, ExAC, and 1000 Genomes, or in disease-causing mutation databases such as HGMD, ClinVar, and LOVD. In silico analyses suggested that these two variants were potentially pathogenic (Additional file 1: Table S1). The alignment of the GCDH sequences revealed that amino acids at positions 37 and 339 were strictly conserved (Additional file 2: Fig. S1). The frameshift variant c.108_109delAC (p.Gln37Glufs*5) would produce a truncated protein that leads to nonsense-mediated decay. According to the UniProt database analysis, the termination of the protein at position 41 affected the functional domains of GCDH, including N-terminal (protein position 47–173), acyl-CoA dehydrogenase, central domain (protein position 176–269), and C-terminal domain (protein position 286–432), which may affect the protein structure and function (Fig. 1). Protein modeling showed that the variant c.1016T>C (p.Met339Thr) increased hydrogen bonds between the protein positions 335 and 339, which may affect the structure and function of GCDH by altering the protein folding process (Fig. 2).

## Discussion

Since the first description of GA1 patients in 1975, over 600 individuals with GA1 have been documented to date [3]. The birth incidence of GA1 varies between 1:30,000 and 1:100,000 newborns; however, a much higher prevalence (1:200 to 1:2300) has been reported in certain groups, such as the Old Order Amish, Irish Travelers, Canadian Ojibway-Cree Indians, and black South Africans [9]. The exact incidence of GA1 in China remains unclear. The first multicenter pilot study revealed that the incidence was 1 in 185,971 newborns in mainland China [10]. In recent years, an increasing number of GA1 patients have been detected with NBS because of increased screening experience and improved genetic diagnostics [11, 12]. The incidence of GA1 in the selected southern Chinese population was approximately 1 in 47,044 births, similar to other studied populations [13, 14]. Compared with the incidence reported in other Chinese regions, the local incidence is higher than that reported in the Zhejiang (1:64,708) and Jiangsu (1:89,335) provinces of southern China [15, 16] and Xi’an City (1:73,076) in northern China [17].

All 13 GA1 patients in this study were identified through NBS. Interestingly, three patients were diagnosed with GA1 due to the low C0 levels during NBS, rather than the typical elevation of C5DC levels, including one neonatal GA1 and two maternal GA1 patients. The neonatal GA1 patient with a low C0 level is suggestive of primary carnitine deficiency (PCD). However, no *SLC22A5* genetic variants were detected in the patient. We speculate that this low C0 level is most likely maternally derived; however, the mother refused to undergo further investigation and therefore, a valid conclusion could not be drawn. To the best of our knowledge, this is the first report of neonatal GA1 with low C0 levels diagnosed through NBS. In comparison, both maternal GA1 patients exhibited typical elevated C5DC levels, although with low C0 levels that were significantly different from those of newborns. Therefore, the presents revealed that few neonatal GA1 patients might have atypical acylcarnitine profiles that are easy to miss when doing NBS.

Although NBS can effectively identify GA1 and is a cost-effective diagnostic strategy [18], false-negative results may occur. Several missed cases have been reported when doing NBS due to various factors [8, 13, 19–21]. The most common factor was that GA1 patients with the LE phenotype and normal C5DC levels would escape detection, as evidenced by one of our patients (no. 7) whose C5DC level was far below the cutoff upper limit. Unlike the previously reported cases [8, 13, 20, 21], the patient had persistent low C0 levels and was detected accidentally. The patient would not have been correctly diagnosed if targeted exome sequencing had not been conducted, indicating the importance of performing multigene panel testing in newborns with low C0 levels. This special case reminders us that an adequate level of C0 is a prerequisite for judging the NBS results, more attention should be paid to the interpretation of NBS testing at low carnitine levels. Another factor that led to missed cases was that GA1 patients initially had elevated C5DC levels but normalized with the second screen, which may be caused by secondary carnitine depletion. In addition, inadequate setting of cutoff values or laboratory interpretation errors were responsible for some missed cases.

Adult-onset GA1 is extremely rare. Only a few adult cases have been reported to date, and most of them were asymptomatic female patients who were incidentally diagnosed due to the abnormal NBS results of their infants [22–25]. This study described two asymptomatic maternal GA1 patients with detailed acylcarnitine profiles, which improves our understanding of this disorder. The identification of maternal GA1 patients is an additional benefit because these seemingly asymptomatic mothers may develop neurological symptoms, and preventive metabolic therapy can prevent neurological deterioration.

At least 240 pathogenic *GCDH* variants have been reported thus far, although several common pan-ethnic pathogenic variants have also been identified (http://www.hgmd.cf.ac.uk). In particular, c.1204 C>T (p.Arg402Trp) was found to be highly prevalent in several populations, with frequencies of 12–40% in western Europe, 57% in Spain, 50% in Poland, 18.8% in India, and 56.38% in Russia [9, 26]. However, the variant was absent in Japan and was rarely identified in both this cohort and previously reported Chinese patients [27–29], indicating ethnic specificity. The c.1244-2 A>C splice site variant destroys the canonical splice acceptor site in intron 11 of the *GCDH* gene and is expected to cause aberrant splicing. Although c.1244-2 A>C variant was reported as the common variant in southern Chinese origin cohorts, it was not prevalent in other Chinese groups [30–32]. Consistent with previous studies [30, 31], c.1244-2 A>C was the most common *GCDH* variant detected in this cohort of patients, with a high allelic frequency of 54.55%. Thus, the *GCDH* variant spectra vary significantly between ethnicities, which may also differ among different populations in the same ethnic group. The *GCDH* variant spectra in this study were generally heterogeneous, except for the observed recurrent variant. The novel variants identified in this study expanded the *GCDH* variant spectra. Combining the *in silico* prediction tools with protein modeling analysis can further reinforce the reliability of the prediction results.

## Conclusions

In summary, this study investigated 13 Chinese GA1 patients identified through NBS. The incidence of GA1 in the selected population was estimated to be 1 in 47,044 newborns. The c.1244-2 A>C variant was the most common *GCDH* variant. Novel variants further expanded the *GCDH* variant spectra. Neonatal GA1 patients with increased C5DC levels can be identified through NBS, maternal GA1 patients can also be detected during NBS due to the low infant C0 levels. Few neonatal GA1 patients may have atypical acylcarnitine profiles that are easy to miss with NBS. Thus, multigene panel testing should be performed in newborns with low C0 levels.

## Materials and methods

### Newborn screening and laboratory tests

From January 2014 to September 2020, a total of 517,484 newborns (292,880 males and 224,604 females) were screened using MS/MS in the NBS center at Quanzhou Maternity and Children’s Hospital and were recruited for this study. Blood samples were collected via heel prick 3 to 7 days after birth and blotted on Whatman 903 filter paper. Dried blood spot (DBS) samples were delivered by cold-chain transportation to our NBS center within three days. DBS samples were pre-processed and then quantified using a mass spectrometer, and internal and external quality controls were performed as previously described [33]. Newborns with elevated C5DC levels were called back. Patients who tested positive with the second screen were referred for auxiliary tests, including biochemical laboratory tests (blood glucose, ammonia, ketones, liver and renal function, serum electrolytes, creatine kinase, and blood gas analysis), and urinary organic acid analysis. Genetic analysis was performed as a confirmatory test. This study was approved by the Ethical Committee of Quanzhou Maternity and Children’s Hospital. Written informed consent was obtained from the parents of all patients.

### Genetic testing and variants analysis

Genomic DNA was isolated from peripheral whole blood using the TIANamp Blood DNA kit (Tiangen Biotech, China), following the manufacturer’s instructions. Targeted next-generation sequencing (NGS) was performed by the Hangzhou Biosan Clinical Laboratory Co. Ltd. (Hangzhou, Zhejiang, China), as previously described [34]. Targeted NGS was performed on the probands using a target sequencing panel of 94 genes (including *GCDH* and *SLC22A5*) associated with inherited metabolic disorders (Additional file 3: Table S2). The coding exons were captured using an Agilent High Sensitivity DNA Kit (Agilent, Santa Clara, CA, USA). The captured fragments were sequenced using the Illumina NextSeq 500 platform (Illumina Inc., San Diego, CA, USA) in paired-end mode, generating 150-bp paired-end reads. The paired-end reads were quality trimmed using the Trimmomatic program (http://www.usadellab.org/cms/index.php?page¼trimmomatic) and aligned to the human genome reference sequence (UCSC Genome build hg19). Single-nucleotide polymorphisms (SNPs) and insertions or deletions were identified using the SAMtools software (http://www.htslib.org/). All possible disease-causing variants identified by NGS were further validated through Sanger sequencing of the patients and their parents. The *GCDH* exons were amplified using polymerase chain reaction (PCR). Amplified fragments were sequenced directly using an ABI Prism 3500 automatic sequencer (Applied Biosystems, Foster City, CA, USA). PCR primer sequences and protocols are available upon request. The pathogenicity of novel variants was assessed using several *in silico* tools, including SIFT, PolyPhen-2, PROVEAN, and MutationTaster. Evolutionary conservation was analyzed using ClustalX (http://www.clustal.org/clustal2). To build three-dimensional (3D) models of GCDH, homology modeling was employed using the Swiss Model Workspace with the PDB accession number Q92947, and PDB files were then submitted to Swiss-Pdb Viewer 4.10 for 3D-structure analysis.

## Supplementary Information

**Additional file 1: Table S1.** In silico prediction and analysis of the novel *GCDH* variants identified by our team..

**Additional file 2: Fig. S1.** Multiple sequence alignment using ClustalX. The amino acid residues at positions 37 and 339 in the GCDH protein (highlighted in box) are strictly conserved among various species.

**Additional file 3: Table S2.** The list of targeted genes.

## Data Availability

The datasets used and/or analysed during the current study can be obtained from the corresponding author upon a reasonable request.

## Abbreviations

GA1: Glutaric acidemia type 1
GA: Glutaric acid
3HGA: 3-Hydroxyglutaric acid
MD: Movement disorders
LE: Low excretor
HE: High excretor
C5DC: Glutarylcarnitine
MS/MS: Tandem mass spectrometry
NBS: Newborn screening
DBS: Dried blood spot
NGS: Next-generation sequencing
PCR: Polymerase chain reaction
3D: Three-dimensional
C0: Free carnitine
PCD: Primary carnitine deficiency
