# Supplementary material for: Biochemical and molecular features of Chinese patients with glutaric acidemia type 1 detected through newborn screening
Source: Orphanet J Rare Dis. 2021 Aug 3;16:339. doi: 10.1186/s13023-021-01964-5 (PMC8335863; doi:10.1186/s13023-021-01964-5)
Supplement: Supplementary file 1 — Additional file 1: Table S1. In silico prediction and analysis of the novel GCDH variants identified by our team.. [file 13023_2021_1964_MOESM1_ESM.docx]

**Table S1.** *In silico* prediction and analysis of the newly *GCDH* variants identified by our team

| No. | Location | Nucleotide change | Protein change | SIFT^a^ | PolyPhen-2^b^ | PROVEAN^c^ | Mutation Taster^d^ | HGMD^e^ | ClinVar^f^ | LOVD^g^ | dbSNP^h^ | Freq in  GnomAD^i^ | Freq in ExAC^i^ | Freq in 1000 Genome^j^ |
| --- | --- | --- | --- | --- | --- | --- | --- | --- | --- | --- | --- | --- | --- | --- |
| 1 | Exon 2 | c.108_109delAC | p.Gln37Glufs*5 | N/A | N/A | -6.047 | 1 | ND | ND | ND | ND | ND | ND | ND |
| 2 | Exon 9 | c.1016T > C | p.Met339Thr | 0 | 0.996 | -4.60 | 0.999 | ND | ND | ND | ND | ND | ND | ND |

ND: no data.

N/A: not available.

^a^SIFT: <http://sift.jcvi.org/>, ^b^PolyPhen-2: <http://genetics.bwh.harvard.edu/pph2/>, ^c^PROVEAN: <http://provean.jcvi.org/index.php>, ^d^MutationTaster: <http://www.mutationtaster.org/>, ^e^HGMD: <http://www.hgmd.cf.ac.uk/ac/index.php>, ^f^ClinVar: <https://www.ncbi.nlm.nih.gov/clinvar/>, ^g^Leiden Open Variation Database <http://www.lovd.nl/3.0/home>, ^h^dbSNP: <https://www.ncbi.nlm.nih.gov/projects/SNP/>, ^i^GnomAD/ExAC: <http://gnomad.broadinstitute.org/>, ^j^1000 Genome Project: http://www.1000genomes.org/.
