# Supplementary figures and images for: Biochemical and molecular features of Chinese patients with glutaric acidemia type 1 detected through newborn screening
Source: Orphanet J Rare Dis. 2021 Aug 3;16:339. doi: 10.1186/s13023-021-01964-5 (PMC8335863; doi:10.1186/s13023-021-01964-5)

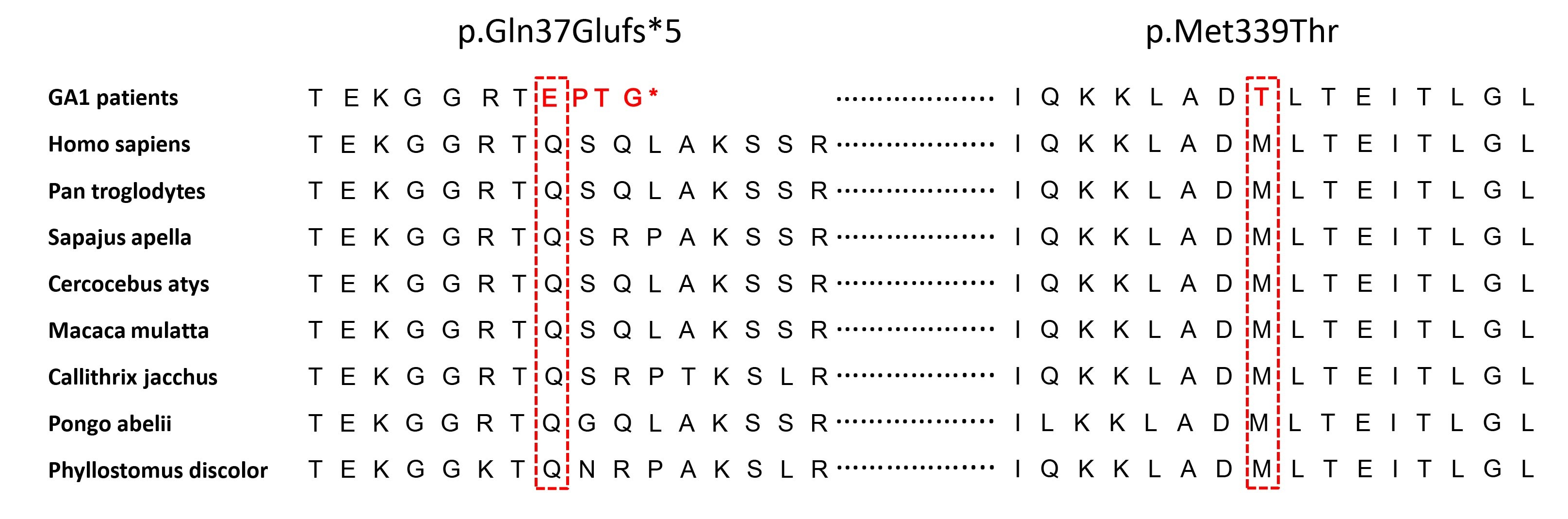

Supplement: Supplementary file 2 — Additional file 2: Fig. S1. Multiple sequence alignment using ClustalX. The amino acid residues at positions 37 and 339 in the GCDH protein (highlighted in box) are strictly conserved among various species. [file 13023_2021_1964_MOESM2_ESM.tiff]
