# Supplementary material for: Biochemical and molecular features of Chinese patients with glutaric acidemia type 1 detected through newborn screening
Source: Orphanet J Rare Dis. 2021 Aug 3;16:339. doi: 10.1186/s13023-021-01964-5 (PMC8335863; doi:10.1186/s13023-021-01964-5)
Supplement: Supplementary file 3 — Additional file 3: Table S2. The list of targeted genes. [file 13023_2021_1964_MOESM3_ESM.docx]

**Table S3.** The list of targeted genes

| Number of genes | Targeted genes |
| --- | --- |
| 94 | *PAH, PTS, PCBD1, QDPR, SPR, GCH1, BCKDHA, BCKDHB, DBT, DLD, AMT, GCSH, GLDC, MAT1A, CBS, CTH, MTHFR, SUOX, MOCS1, MOCS2, GPHN, FAH, TAT, HPD, HGD, MUT, MMAA, MMAB, MMACHC, MMADHC, LMBRD1, ABCD4, MCEE, CD320, MLYCD, SUCLA2, SUCLG1, SUCLG2, PCCA, PCCB,* ***GCDH****, IVD, BTD, ACADSB, AUH, DNAJC19, CLPB, TMEM70, SERAC1, HMGCL, MCCC1, MCCC2, HLCS, ACADS, ACADM, ACADVL, HADH, HADHA, HADHB, ACAD8, ETHE1, ETFA, ETFB, ETFDH, ACAT1,* ***SLC22A5****, SLC25A20, CPT1A, CPT2, ARG1, ASL, ASS1, SLC25A13, CPS1, OAT, OTC, SLC25A15, SLC5A5, TPO, TG, TSHB, TSHR, PAX8, DUOX2, CYP11B1, CYP11B2, HSD3B2, STAR, CYP17A1, CYP11A1, POR, G6PD, ATP7B, PC* |
